# Russia between east and west, and the future of Eurasian order

**DOI:** 10.1057/s41311-020-00261-5

**Published:** 2020-07-14

**Authors:** Elena Korosteleva, Zachary Paikin

**Affiliations:** University of Kent, Canterbury, UK

**Keywords:** Russia, Central Eurasia, Order, ‘the local’, Complexity, China, EU

## Abstract

This article introduces the special issue by going beyond the traditional debates about geopolitics and great power rivalry. Instead, it examines the emergent and highly complex world of Central Eurasia, in its transformative effort to reorder itself in response to both global and local change. In particular, the paper (and the volume) focuses on two interrelated themes: one of a *changing Russia*, that is anxiously trying to adapt to the uncertain dynamics within and beyond the wider Eurasian space; and the other—of an emerging complexity of new order-making regional (integration) initiatives that are poised to reshape the future of international and global order. The overarching intention of this paper and the volume is to advance the need to focus on ‘the local’, to gain a more holistic understanding of the present-day challenges and the kind of global response needed to stay attuned to the increasingly complex world.

## Introduction to special issue of *International Politics*

The years since the onset of the Ukraine crisis have seen Russia’s increasing effort to deepen its strategic partnership with China and accelerate its declared ‘pivot to the East’: As Viacheslav Nikonov, Duma parliamentarian and a founder of Russkiy Mir, poignantly noted, Europe was only mentioned once at the 2019 International Economic Forum in St Petersburg.^1^ These developments follow a quarter-century of rising disputes between Russia and the West that were nonetheless characterized by a nominal commitment to construct some sort of ‘Greater Europe’ from Lisbon to Vladivostok rooted in common economic, political, security and cultural spaces. The Euromaidan protests saw Ukraine caught between two competing deals and regulatory orders—put forward respectively by the European Union (EU) and the fledgling Eurasian Economic Union (EAEU)—that were ultimately not reconciled. Ukraine has since placed itself on a path of ‘Europeanization’ and Western integration, concluding an Association Agreement (AA) and Deep and Comprehensive Free Trade Area (DCFTA) with the EU while enshrining its desire to join NATO and the EU in its national constitution.^2^

This process has coincided with the launch of Moscow’s ‘Greater Eurasia’ initiative, which officially seeks to promote *pan*-*Eurasian* integration without sacrificing Russia’s sovereign decision-making or notional equality in international affairs (Karaganov 2016; Trenin 2019a). This split between Russia and Ukraine, which were largely part of a single polity for several centuries, leaves the former’s *European* future and the boundaries of its national community uncertain. Furthermore, it is also associated with an invisible ‘loyalty’ rupture and a deepening crisis of relations between Russia and its immediate neighbourhood manifest in the recent disputes with Belarus regarding the future of the Union State,^3^ Azerbaijan’s reluctance to engage Russia in its connectivity talks with China and the EU, or Uzbekistan’s sudden decision to forgo EAEU membership, settling on observer status instead.^4^ Russia has thus been left stranded to a certain extent between East and West in an era of supercontinent-wide integration projects—ranging from China’s Belt and Road Initiative (BRI) to the EU’s Asia connectivity strategy.

The above dynamics also need to be situated in the context of deep and pervasive change observable regionally and globally, including China being on the cusp of global influence and, some would argue, global disaster owed the coronavirus outbreak^5^; the Trump administration becoming more erratic after the president’s acquittal by the Senate in his impeachment trial; and the EU finding itself at a crossroads of history in a post-Brexit environment with new (centrifugal) internal and external power dynamics. The overarching picture is that of turmoil, growing uncertainty and complexity, which the EU’s Global Security Strategy back in 2016 described as ‘predictable unpredictability’ (EEAS 2016) occurring in a world of increasing contestation between the established liberal and newly emerging (potentially rival) orders (Flockhart 2016).

Our ambition in this special issue is to take stock of these complex developments and go beyond the existing debates that traditionally focus on geopolitics and great power rivalry so well-rehearsed in the mainstream scholarship over the past century. Instead, we wish to take our discussion a step further and look at the future of the emergent and highly complex world of Central Eurasia,^6^ that is reordering itself to respond better to both global and local change, in a shared effort of (re-)learning to cooperate and cohabitate. In particular, we will focus on two interrelated themes, to conceptualize a more holistic understanding of the emerging challenges: one of a *changing Russia*, that is anxiously trying to adapt to the uncertain dynamics within and beyond the wider Eurasian space, and sitting awkwardly, once more between east and west, while defining its own future; and the other—of an emerging complexity of new order-making regional (integration) initiatives that are poised to reshape the future of international and global order. The overarching argument this special issue seeks to advance is that of the need to drill into ‘the local’ and domestic, to gain a more holistic picture of the present-day challenges and the global governance response needed to stay better attuned to the increasingly multiplex (Acharya 2017) and multi-order (Flockhart 2016) world.

The special issue is a result of ongoing research and debates between and beyond the contributors to this volume working on the aforementioned themes. It stems from discussions held at conferences and workshops, including at LSE IDEAS^7^ brought together by two projects—the H2020 UPTAKE and GCRF COMPASS^8^; the GCRF COMPASS BRI @5 workshop held at Kent and Cambridge^9^; and especially the GCRF signature conference on Governance and Resilience in wider Eurasia, held in July 2019, which brought together scholars from Europe, China, Russia, and Central Asia, to reflect the developments across an increasingly central Eurasian space.^10^

This introduction will proceed by unpacking the two core themes of the volume—a *changing Russia,* between east and west once more; and a *rising Eurasia*, shaping a new cooperative order(s) and global futures, to be followed by a brief account of individual contributions to explicate the analysis further.

## A changing Russia, stranded between east and west, once more?

Russia’s desire to ‘join’ Europe has taken various forms throughout history. Most notably, following the ‘gathering of the Russian lands’ that occurred after the throwing off of the Mongol yoke, Tsar Peter the Great set the country on a path of perceived modernization and Europeanization, including the transfer of the national capital to St. Petersburg on the Gulf of Finland. According to most conventional narratives, Russia had been fully incorporated into the European balance of power system by the mid-eighteenth century, around the time of the Seven Years’ War (Watson 1984:70–71; Kissinger 2014:31–59). However, one should not confuse this expression of Europeanness with a desire for uniformity, as the desire to ‘catch up’ with the West at times owed more to a pressing need to modernize militarily and technologically rather than adopt Western political values (Watson 1984:63; Porter 1996:125–127). Russia’s situation at Europe’s periphery renders its relationship with the continent complex. It has competed for some time with—among other varied intellectual tendencies such as nationalism and pan-Slavism—a strain of Eurasianist thinking that has emphasized Russia’s distinctness from Europe, although this has taken differing forms: highlighting the need for a partnership with China or to defend a distinct Eurasian space separate from the rest of Asia from China; embracing Russia’s multi-ethnic character or rejecting it in favour of more explicit leadership by ethnic Russians (Laruelle 2017:156). There has also been speculation as to whether Eurasianism can succeed as a cultural model or ideal on a par with the appeal of European civilization (Rozman 2014:202).

This tug of war between Europhile and Eurosceptic tendencies in Russian politics and foreign policy has played out over the past several centuries. The end of the Cold War was expected to presage Russia’s ‘return to Europe’ after having been cut off for several decades by the Iron Curtain. And although various disputes emerged over the norms and practices that should underpin the global and European orders (Stent 2014; Hill 2019), even after the Ukraine crisis Russia has been keen to stress that the EAEU and its vision of a ‘Greater Eurasia’ aim to be complementary to both the EU and the global liberal trading architecture (Karaganov 2016; Sakwa 2017:147–150). Vladimir Putin entered the Kremlin at the start of the century aiming to repair much of the relationship with the West that had been damaged after the NATO intervention in Kosovo but has presided over a period in which this relationship has further deteriorated. As such, much of the rhetoric he has employed has sought to balance between support for and criticism of norms, values and institutions that are traditionally associated with the West and with the so-called ‘liberal international order’ (Zagorski 2008:47; Malinova 2012:77–82).

The situation is further complicated by the effects resulting from the dissolution of the Soviet Union. It is often forgotten that the agreements and dynamics that brought the Cold War to an end occurred before the USSR’s collapse. Notions surrounding a declared Russian ‘sphere of privileged interests’ and ‘near abroad’ suggest that Moscow had not fully internalized the implications of the Soviet Union’s collapse in the early 1990s. The precise meaning of the non-Russian republics’ sovereignty and independence was not immediately obvious (Plokhy 2015), and it wasn’t until after the start of the war in the eastern Donbass that the ‘loss’ of Ukraine became to a certain extent clear (Trenin 2018). As such, for the first time in several centuries, post-Maidan Russia is ‘just’ Russia (Trenin 2019b).

The partial shift in Russian foreign policy away from Europe towards an emphasis on Eurasia has been accompanied by changes in Russian domestic politics as well. While Russia’s political system does not resemble the Leninist institutions of China, shared authoritarian concerns and scepticism surrounding ‘colour revolutions’ has acted as a lubricant in facilitating a normative rapprochement in Sino-Russian relations in opposition to Western interventionism and democracy promotion (Paikin et al. 2019). Perhaps more crucially, it has become increasingly clear as the twenty-first century has progressed that Russia has not been able—and perhaps even will not be able—to transform itself into a capitalist liberal democracy on a par with Western states. This has been illustrated by references to ‘Putinomics’, a form of economics that embraces elements of the free market without transforming Russia into a full-fledged market economy, as well as through Putin’s alleged desire to ‘build an alternative’ to the Western economic and political consensus (Baunov 2018). Recent constitutional moves to strengthen the Russian Duma at the expense of the presidency could be interpreted as efforts by Putin to strengthen the resilience of the current system’s political institutions, having acknowledged that a complete transition to a Western-type set-up is not possible. This builds upon Putin’s now-infamous recent comment that liberalism has become ‘obsolete’ (Barber and Foy 2019), perhaps more reflective of the frustration felt by many in the Russian elite surrounding their country’s inability to join the West than of the populist challenge currently being faced in many Western societies (Liik 2019). Moreover, even if the Russian political system does undergo a profound change beyond the expiration of Putin’s current presidential term in 2024, it could be that China has become too powerful and the Sino-Russian relationship too developed for Moscow to turn its back on Beijing and fully ‘return to Europe’, as it were. This will particularly remain the case so long as rival visions prevail with respect to how to order the wider European space, with Western capitals’ insistence on the right of Eastern European states to choose their geopolitical orientation remaining seemingly incompatible with Moscow’s proclaimed desire for an ‘equal relationship’ with the West.

As such, Russia remains seemingly stranded between East and West, unable to capitalize on its natural synergies with Europe but likely reduced to second-tier status in the emerging Asia–Pacific security system (Kortunov 2019). And yet, despite the growth of Eurasianism in contemporary Russian political discourse, the notion of Europe and the broader West as representing Russia’s perennial ‘Other’ image remains strong, with some scholars going so far as to posit a ‘subaltern’ normative-discursive dependency (Morozov 2015). As a result, while it might be contended that political and values-related differences between Russia and Western Europe will impede the development of an intimate or ‘convergent’ international relationship between them (Buzan 2012:38–45), it may be Russia’s simultaneous illiberalism and Europeanness that represents the driving force in the Russia-Europe split rather than any emergent Eurasianism. In other words, Russia’s contention that it belongs to the European ‘family of nations’ without upholding the liberal principles enshrined in the Paris Charter of 1990—often thought of as the document that ended the Cold War and launched a vision of a Europe ‘whole and free’—inherently challenges the Western European notion of what it means to be European. This adds an additional layer of complexity underpinning Russia’s ambiguous position between East and West, and the resulting ideational contestation that it has produced ensures that the principles underpinning the fledgling pan-Eurasian order will remain a matter of contention.

The result of all this is, in many ways, an uncertain and novel picture: Russia remains somewhat influenced by the liberalism and Westernism of the 1990s even as it gradually adopts a more conservative and populist discourse rooted in ‘strong social and patriotic rhetoric […] family, order, [and] spirituality’ (Stoyanova 2019); a country with a lengthy history but nonetheless a fresh polity forced to chart a new path. Even the various orientations to which Russia is prone—liberal, Eurasianist, etc.—can take multiple forms (Sergunin 2004). The country’s accelerated ‘pivot to the East’ is also multifaceted and perhaps the most serious attempt that Moscow has made in the post-Cold War era to engage deeply with Asia and Eurasia (Lo 2019). Its grand projects vary greatly in nature, from the geographically bounded EAEU to the more expansive vision of a Greater Eurasian partnership from Lisbon to Shanghai. There is a nominal desire to pursue integration across the board and relations with China in particular are deepening on both economic and security issues, but the recently signed trade agreement between the EAEU and China is non-preferential, suggesting a more cautious approach. And although Russia’s sprawling geography gives it the ability to project power into a whole host of regions, its interests vary from place to place—the regular reminder that Central Asia lies within Russia’s traditional sphere of influence contrasts sharply with Moscow’s more restrained behaviour and rhetoric in the Far East and Northeast Asia.

While the desire to be treated as an equal great power has been a recurring theme for Russia in the post-Cold War era, the initial desire to create a ‘Greater Europe’ from Lisbon to Vladivostok and the more recent deepening strategic partnership with China together indicate that the EAEU alone is insufficient to guarantee Moscow continued great power status at the global level over the long term. Still, the ability to project (at the very least) disruptive power with great efficacy demonstrates that Russia retains the ability to impact the shape of order across much of Eurasia, from Europe, to the Middle East, to Central Asia and beyond. The articles in this special issue aim to contribute to the conceptualization of the nature of Russia’s impact on the evolving (perhaps, in some ways, fledgling) Eurasian order as the country continues along its extended period of transition, reflecting on the past and present to provide a potentially clearer picture of the future.

## A *rising Eurasia* and the future of regional and global orders

This focus on Eurasia in particular owes itself to recent developments that have been accumulating over the past several years and decades. While the relative importance of dominating the Eurasian ‘Heartland’ has long been a concern of geopolitics, focus on the challenges facing the so-called ‘liberal international order’ has hitherto caused much analysis to be focused on the *global level* (see for example, Ikenberry 2018; Acharya 2018; Lebow 2018; Buzan and Schouenborg 2018; Reus-Smit and Dunne 2017; Sørenson 2016). And while questions surrounding the rise of regionalism have featured prominently in the field of international relations since the end of the Cold War, the gradual redefinition of Washington’s global role—taking its fullest form thus far under the Trump administration—and the late rise of Central Asia and the Caucasus (as a gateway to the Middle East) suggest that the future of the Eurasian supercontinent may lie, to a large extent, in the hands of its indigenous (and yet previously neglected) actors, and a need to shift to *the local level*, to understand better the emergent ordering dynamics there. It is instructive to observe how the microcosm of domestic relations and their external projections by local actors—e.g. Kazakhstan as evidenced in Pieper’s article, or Azerbaijan as referred to by Valiyev et al., or indeed the EAEU difficult integration dynamics as discussed by Korosteleva and Petrova in this volume—impact the hitherto seemingly ‘stable’ great powers’ status-quo vis-à-vis the region. Notably, Russia’s recent assertiveness has emphatically coincided with the perceived further consolidation of the European Union and its extended outreach to the ‘neighbours of the neighbours’ following the launch of a new EU Strategy towards Central Asia,^11^ as well as with the initiation of China’s BRI effectively targeting the same space.^12^ And while much of the EU’s continued dependence on American security guarantees may alter the shape of Eurasian integration, the launch of the EU’s Asian connectivity strategy indicates a willingness to respond in some form to China’s entreaties, even if the norms and standards surrounding these projects may remain a matter of contestation, as argued by Lukin, Paikin and Nitoiu et al. in this volume. To this end, the EU Global Security Strategy (2016) explicitly reflected on the need to develop cooperative orders across the region and beyond; and yet, it proves difficult to put it to practice as posited by Korosteleva and Petrova in this special issue. Furthermore, Sakwa’s contention (2016, and in this volume) that a ‘clash between norms and spatiality’—between clashing regulatory and political orders and the reality of countries’ geographic location—was largely to blame for the onset of the Ukraine crisis may suggest that a focus on ‘spatiality’ and the role of indigenous and (order-making) actors in shaping that space is imperative, with implications for understanding international and global orders.

Engaging with this new imaginary of Eurasia brings the need to redefine the term *Central Eurasia* as a new emergent ordering locality, with a global appeal. This special issue treats the Eurasian space as an evolving construct consisting of several ‘nested’ identities, which was reflected by a series of contributions to this volume. The space has been traditionally viewed as a rough location where Eurasia’s three principle powers—the EU, Russia and China—and other actors are jockeying for material and normative influence. It is in this broad region where the Russian-backed EAEU is attempting to consolidate its presence, and also where several of the corridors of the BRI connecting Asian and European markets lie. It is defined by a mixture of collaboration and contestation between actors, ranging from the nominal promise made by Russia and China to harmonize and coordinate the EAEU and BRI projects, to the EU’s cautious embrace of the BRI so long as it adheres to a list of economic, environmental and other standards.

At the same time, the translation of this globality of wider EU-Asia interaction into the locality of many powerful and still emergent actors has begun to shape and challenge the status quo of the traditional space for great power politics. And although much of the future of global affairs will also be determined by events in the ‘Indo-Pacific’ or Asia–Pacific or indeed African regions, the confluence of the emergent indigenous players in Central Eurasia—all of which are in transition in various ways—on issues such as transport, institutions, security, culture, religion and the philosophy of being (*hamsoya*, meaning life in the shadow of your neighbour)—that comes to designate this region as a composition of smaller entities forming a system of a rising collective ‘player’, with many voices, aiming to affect the supercontinent-wide and global equilibrium. It is in this locality that many contributions of the volume view Central Eurasia—as a region roughly spanning Belarus in the west, Afghanistan in the south, and Mongolia in the east—an immensely rich space, a configuration of domestic politics, and intra- and inter-regional dynamics, presently contested by the EU, Russia and China, that comes to set its own voice and ordering arrangements.

This is why discussing the future of Eurasian order(s) requires one to engage with both ‘the local’ as well as what constitutes an *international and even global order*, as argued by Haukkala’s and Paikin’s articles in this volume. Lebow (2018:305–306) defines order as ‘legible, predictable behaviour in accord with recognized norms’ that requires a degree of solidarity to remain robust. From an English School perspective, an international order can range from mere patterns of behaviour to an integral component upholding the stability of an international society of states (Reus-Smit and Dunne 2017:31–33). On the other hand, order could also be viewed as a very local effort to turn hitherto fragmented communities into powerful ‘peoplehood’, making them more ambitious in a strife for good life and resilient in the face of adversity, drawing on their ‘inner strength’ and future aspirations (Korosteleva and Flockhart 2020). This kind of ‘local order’, if shared through cultural affinity, language, religion and philosophy of being, could challenge any geographical border (especially in a nomad culture), and shape the future of Eurasia in another, bottom-up and potentially more sustainable way.

This wide range of definitions is useful, as the term ‘order’ could be interpreted as requiring stability or alternatively as merely representing the status quo at any given time. Tang, for example, argued that order, if anything, should be defined as a ‘degree of predictability of what is going on within a social system’ (Tang 2016:32). This certainly makes sense, when applied to local dynamics within the complex system of Eurasia, to understand what is likely to emerge in the future as part of the Eurasian space.

In other words, as this volume attests to through its various contributions—examining the emergence and reshaping of Eurasia, bottom-up, horizontally through various integration initiatives, and top-down via the still dominant great power politics—contestation is now an essential and constitutive part of any order and its maintenance. What matters is to stay attuned to internal dynamics and be responsive to change in an adaptive way (Flockhart 2020) which would necessitate not just the emergence but cohabitation of new orders, to be potentially hosted by a complex Eurasian space. With contestation between the EU and Russia in the eastern neighbourhood having become particularly apparent in recent years, contrasting with the mixed rapprochement and joint Sino-Russian attempts at regional stabilization in Central Asia, exploring the foundations of the existing and emergent order(s)—particularly in Central Eurasia—has become an important and timely task. This debate over contestation and order juxtaposes itself with questions surrounding the nature and facets of *resilience* in the international order, covered by several articles in this issue as well. These questions are particularly of conceptual interest, as they shed light on how processes of change affecting an international order can—perhaps counterintuitively—indicate elements of its robustness, durability and strength.

## Contributions to the special issue

Individual contributions to this special issue are structured in a particular way to expand the conceptual debate that currently surrounds the nature of order—be it local, regional or global—and the role of traditional great power politics in it. On the other hand, many contributions focus on empirical dynamics and local response, to demonstrate how traditionally understood power politics is challenged today by the emergent complexity and the increasing unpredictability of global politics. What makes this volume special is the richness of its outlook, and the coherence of the message posited to the reader—of the need to be responsive to change, both in practice and thinking, in order to be able to imagine a more sustainable future, of diverse and cooperative orders. This special issue challenges the traditional conceptual undertakings and brings together a critique of historical, political and IR debates, with a focus on Central Eurasia, and its present (re)ordering, with local and global implications.

To provide some initial context surrounding Russia’s place in the post-Cold War order, the volume begins with Sakwa’s discussion of the country’s complex relationship with the West, further developing the notion advanced in his 2017 book that Russia is neither a status quo power nor a revisionist one but rather ‘neo-revisionist’, challenging the perceived excesses of the Western-led liberal international order but defending the integrity and autonomy of global international society. In particular, Vladimir Putin’s recent comment to the effect that liberalism has become ‘obsolete’ has raised questions concerning whether Moscow has now set out to create an alternative to the Western model and is challenging the existing global order outright. Sakwa notes that Putin’s critique contains both a cultural and a geopolitical element, and that the opposition is not to the liberal order per se but rather to the notion that the spread of liberal order can be equated with the spread of order full stop. This places Russia in an ambiguous conceptual position between east and west as it continues to chart its post-Cold War identity.

The special issue continues with three analyses that seek to provide further specifics of Russia’s current situation between east and west. Lukin and Paikin in two separate articles focus on the specifics of the Sino-Russian relationship and the extent to which the two countries can forge a deep and durable partnership. Lukin focuses on the prospects of a Sino-Russian alliance and delves into the areas of Eurasia where Moscow and Beijing possess intersecting interests, while Paikin revisits the English School theory to posit the extent to which Russia and China are jointly able to construct a ‘thick’ regional international society in Central Eurasia. Haukkala, for his part, turns towards Europe, allowing this special issue to provide a more fully rounded discussion of some of the central regional systems within the emerging pan-Eurasian order. The conclusions that he reaches surrounding Europe’s possible ‘nonpolarity’—i.e. the inability of powers to uphold regional order collectively—provide further illustration of one of this special issue’s central themes, namely an exploration of the extent to which cooperative orders are possible in wider Eurasia.

This is followed by two articles that delve into this conceptual question. Korosteleva and Petrova examine dynamics related to power, principles and practice to evaluate the projects and posture of the EU, Russia and China vis-à-vis the Central Eurasian region. While acknowledging that each of these leading powers has been reflective to a certain extent when it comes to their respective regional strategies, they all too often attempt to project their own norms and priorities onto local actors without sufficient consideration for indigenous interests and needs. This, in turn, brings implications for the (un)likely resilience of regional order(s) in Central Eurasia and the potency of the cooperative great power relations across the region. Nitoiu and Pasatoiu, complementing this analysis, proceed to compare the Russian, Indian and Chinese interpretations of resilience with that of the EU, which featured the concept prominently in its 2016 Global Strategy. In particular, the non-Western view of order as dynamic provides for a differing understanding from the Western notion that the ‘liberal content’ of today’s global order must be upheld. Finally, the volume concludes with two case studies examining how some of the region’s smaller states self-identify and view their complex interests when faced with the entreaties of the various leading actors from across Eurasia—Pieper providing an analysis of Kazakhstan, and Valiyev and Bilalova examining Azerbaijan. Both articles showcase an almost impossible balancing act that many indigenous actors face today across Central Eurasia when having to withstand the pressure and assertiveness of new integration initiatives, while pursuing their own interests and ‘good life’ aspirations. Paradoxically, this pressure helps them to develop their distinctive Self and encourage self-reliance, which, if not recognized and engaged with, may work to counter the very integration initiatives that great power politics instigates.

This special issue raises many important conceptual questions that speak directly to the world of emergent complexity and ordering dynamics, and to how we understand them in International Relations as a discipline. We hope that by unpacking the issues of order, sovereignty, great power politics, global/local nexus, resilience, complexity and change in this volume, not only do we substantively contribute to the new debates about the future of international order(s) and the place of Russia and indigenous actors in it, but also aim to draw attention to the immediate relevance and essentiality of ‘the local’ and ‘the person’ in defining these orders in their future configuration and resilience. This is what makes Central Eurasia perhaps one of the most interesting cases to observe today—as situated at a crossroads of history, culture, the Silk Road legacy and its effervescent and almost Sogdian^13^ adaptability. However, as is always the case, we must acknowledge that there seem to be more questions asked than answers provided at this point of time. Perhaps the most we can hope to achieve in this special issue is a critical appraisal of change and adaptability in Central Eurasia, illuminating the scope of ongoing transformations at both the local and global levels. We hope that together the contributions can move the field towards more productive and specific engagement with ‘order’ and ‘locality’, as well as global and local processes, that would enrich and reshape our understanding of a broad range of issues that are of key importance to the IR discipline and to the real world in which we live.

In other words, with this discussion we hope to generate the kind of debate that will lead us to a better understanding of the need for a cooperative future, in a world of growing complexity and diminishing control, where the only constant that ever remains is ‘the person’ and their (re)ordering response to change.

## Footnotes

1 For more information see Nikonov’s opening speech at the Minsk Dialogue forum 2019: https://www.youtube.com/watch?v=MpizBgRzwPg.

2 https://eeas.europa.eu/headquarters/headquarters-homepage/4081/eu-ukraine-relations-factsheet_en.

3 https://www.ecfr.eu/article/commentary_unsettled_union_the_future_of_the_belarus_russia_relationship.

4 For the full text see President Mirzoyiev’s nation state address on 24 January 2020 visit: https://president.uz/en/lists/view/3324.

5 See the Economist (2020) ‘Special Report on China’s Belt and Road’, 8 February, pp. 1–12.

6 While the geography of Central Eurasia is discussed later in the text, here we specially refer to the space spanning the borders of Europe in the west, Russia in the east, and China in the South.

7 For more information see https://research.kent.ac.uk/global-europe-centre/2018/12/13/russia-between-east-and-west-and-the-future-of-eurasian-order/.

8 For more information on UPTAKE see https://www.uptake.ut.ee/; and on GCRF-COMPASS—https://research.kent.ac.uk/gcrf-compass.

9 https://research.kent.ac.uk/gcrf-compass/wp-content/uploads/sites/169/2019/02/COMPASS-BRI-workshop-Shorter-draft-programme-for-website.pdf.

10 https://research.kent.ac.uk/gcrf-compass/wp-content/uploads/sites/169/2019/11/COMPASS-Signature-Conference-2019-Final-24-June.pdf.

11 https://www.consilium.europa.eu/en/press/press-releases/2019/06/17/central-asia-council-adopts-a-new-eu-strategy-for-the-region/.

12 China is now working on the launch of BRI 2.0 to specifically promote the ‘people-to-people’ dimension. See Korosteleva & Petrova article in this volume.

13 This refers to an ancient civilization of Sogdians who once populated Eurasia, and who were seen as the true masters of trade and cohabitation, effectively absorbing every culture on their nomadic trajectories of life (Nurulla-Khodjaeva 2017).
